# Recent Advances in Multimodal Nanostructured Bioaerogels for Smart Drug Delivery

**DOI:** 10.3390/polym17223012

**Published:** 2025-11-12

**Authors:** Muhanad A. Abdulsamad, Lujin A. Essa, Rabia Alghazeer, Norah Alkhayyal, Rawan Altalhi, Randah Alghamdi, Esam Bashir Yahya

**Affiliations:** 1 Department of Zoology, Faculty of Science, Sabratha University, Sabratha 00218, Libya; 2 Department of Biological Sciences, College of Science, University of Jeddah, Jeddah 23445, Saudi Arabia; laessa@uj.edu.sa (L.A.E.); ralghamdi@uj.edu.sa (R.A.); 3 Department of Chemistry, Faculty of Sciences, University of Tripoli, Tripoli 50676, Libya; 4 Department of Microbiology, Faculty of Science, Al-Asmarya Islamic University, Zliten 218521, Libya

**Keywords:** nanostructured bioaerogels, drug delivery, biosensing, responsive release

## Abstract

The convergence of nanotechnology and bioaerogels has paved the way for the development of multimodal nanostructured bioaerogels with remarkable potential in smart drug delivery systems. These advanced biomaterials integrate multiple functionalities, including sensing, targeting, and therapeutic actions, to enhance drug efficacy, minimize systemic side effects, and enable real-time monitoring of therapeutic responses. This review provides a comprehensive analysis of the structural design, physicochemical properties, and fabrication strategies of multimodal bioaerogels. It further explores their role in responsive drug delivery, emphasizing stimuli-responsive mechanisms such as pH, temperature, and enzymatic triggers. The incorporation of nanomaterials, including metallic nanoparticles, carbon-based nanostructures, and polymeric nanocarriers, has endowed bioaerogels with tunable porosity, controlled drug release, and bioactive functionalities. Additionally, their application in precision medicine, particularly for cancer therapy, antimicrobial treatments, and tissue engineering, is critically examined. Challenges related to scalability, biocompatibility, and regulatory compliance are also discussed, alongside future perspectives on advancing these bioaerogels toward clinical translation. By integrating interdisciplinary insights, this review underscores the transformative potential of multimodal nanostructured bioaerogels in the next generation of intelligent drug delivery systems.

## 1. Introduction

The field of drug delivery has undergone a transformative evolution, driven by the necessity to enhance therapeutic efficacy, minimize side effects, and optimize patient compliance [1,2]. Conventional drug delivery methods, such as oral administration and intravenous injections, often suffer from drawbacks including poor bioavailability, systemic toxicity, and non-specific targeting, which can lead to adverse effects and reduced treatment effectiveness [3]. In response to these challenges, nanostructured bioaerogels in smart drug delivery have emerged as an innovative approach to precisely control drug release, ensuring that therapeutic agents reach their intended target sites at optimal dosages and at the right time [4]. Unlike conventional or monofunctional aerogels that typically serve a single role, such as passive drug encapsulation or mechanical support, multimodal bioaerogels integrate multiple functional components such as targeting ligands, responsive nanomaterials, and bioactive agents within a single platform [5]. This multimodality enables simultaneous sensing, controlled release, and therapeutic action in response to complex biological cues like pH, enzymes, or temperature changes [6]. In contrast, traditional nanostructured systems often rely on a single mode of action, limiting their adaptability and precision in dynamic physiological environments. Thus, the multimodal design represents a paradigm shift toward smart, adaptive, and multifunctional drug delivery platforms capable of coordinated therapeutic performance. These intelligent systems integrate advanced materials, nanotechnology, and biosensors to enable stimuli-responsive drug release, real-time monitoring, and adaptive therapeutic interventions [7]. Nanostructured bioaerogels in smart drug delivery have been designed to respond to physiological and environmental cues, such as pH [8], temperature [9], enzymatic activity [10], and redox gradients [11], making them particularly promising for applications in cancer therapy, infectious disease treatment, and chronic disease management [12]. Additionally, the integration of targeting ligands and surface modifications allows for improved selectivity, reducing off-target effects and enhancing patient outcomes [10]. Despite their potential, the development of effective nanostructured bioaerogels in smart drug delivery remains a challenge, particularly in terms of optimizing their structural and functional properties to achieve the desired pharmacokinetics and biodistribution.

**Figure 1:**
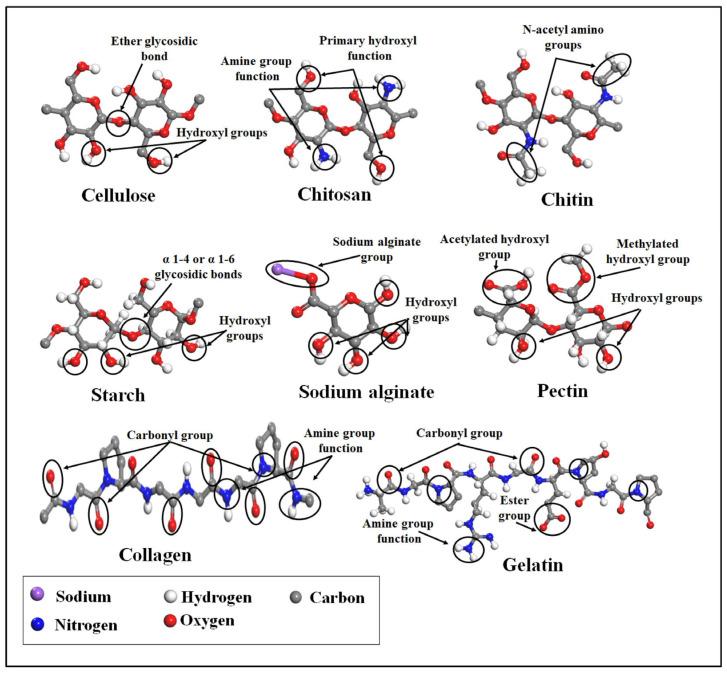
Molecular structures and key functional groups of major naturally derived biopolymers commonly used in biomedical and drug delivery applications. Adapted with permission from [12].

Among the various nanomaterials explored for smart drug delivery, nanostructured bioaerogels have gained increasing attention due to their exceptional porosity, large surface area, biocompatibility, and tunable physicochemical properties [13,14]. Bioaerogels, derived from natural polymers such as chitosan, gelatin, cellulose, alginate, and silk fibroin, provide a sustainable and biodegradable alternative to synthetic carriers [15,16]. Naturally derived biopolymers such as cellulose, chitosan, chitin, starch, sodium alginate, pectin, collagen, and gelatin, etc., have attracted significant attention in biomedical engineering and pharmaceutical formulations due to their biocompatibility, biodegradability, and low toxicity. Figure 1 presents the molecular structures and key functional groups of major naturally derived biopolymers commonly used in biomedical and drug delivery applications, including cellulose, chitosan, chitin, starch, sodium alginate, pectin, collagen, and gelatin. The highlighted functional groups, such as hydroxyl, amine, carboxyl, carbonyl, and ester groups, contribute to their biocompatibility, biodegradability, and ability to form hydrogels or composite matrices for controlled drug delivery and tissue engineering. Their safety for use in drug delivery and medical applications is strongly supported by both biochemical properties and extensive in vivo and clinical evidence [17]. These biopolymers share common safety advantages according to several studies: they are non-carcinogenic, elicit minimal immune responses, and degrade into naturally occurring, non-toxic metabolites [18,19]. Their long history of use in food, pharmaceutical, and medical products supports their regulatory acceptance and clinical reliability. Continuous development of modified and cross-linked derivatives further enhances their functionality while maintaining their excellent biocompatibility profiles, solidifying their position as safe, sustainable materials for advanced drug delivery and medical applications [20].

Their hierarchical porous structure of biopolymeric aerogels enables high drug loading capacity, controlled release profiles, and efficient encapsulation of bioactive molecules [5]. The surface area and interconnected porosity facilitate enhanced drug loading and retention, ensuring efficient encapsulation and sustained release [21]. Additionally, bioaerogels exhibit stimuli-responsive behaviour, allowing for controlled and targeted drug release in response to specific physiological triggers [22]. These materials also offer improved stability and prolonged circulation time, reducing the need for frequent dosing and improving patient compliance [12]. Furthermore, bioaerogels have the potential for multimodal functionalities, integrating biosensing [23], targeting ligands [24], and imaging agents [25], making them suitable for multiple applications.

While substantial progress has been made in the development of bioaerogel-based drug delivery systems, several key gaps in knowledge still persist. Many studies have focused on bioaerogel synthesis and characterization [26,27] or medical applications [28], but comprehensive investigations into their desired properties, mechanism of action in each aspect, and functional performance in smart drug delivery applications remain limited. Additionally, while the potential of bioaerogels for controlled drug delivery is acknowledged, a systematic review integrating their role in sensing, targeting, and therapeutic actions is lacking. This review aims to address these gaps by providing a comprehensive analysis of multimodal nanostructured bioaerogels, emphasizing their integration of sensing mechanisms, targeted delivery, and controlled drug release. Unlike previous reviews that primarily focused on the synthesis or general biomedical use of bioaerogels, the present work uniquely emphasizes multimodal nanostructured bioaerogels that integrate nanotechnology to achieve smart, stimuli-responsive drug delivery. This review highlights the synergistic role of nanomaterials in enhancing the functional versatility, responsiveness, and therapeutic precision of bioaerogels. Furthermore, it provides a critical comparison of design strategies, mechanisms of stimuli-responsiveness, and emerging clinical applications, thereby offering an updated and interdisciplinary perspective on their potential in next-generation intelligent drug delivery systems. By bridging the knowledge gap between material design and functional performance.

## 2. Biocompatibility and Non-Cytotoxicity of Nanostructured Bioaerogels

Biocompatibility is a critical requirement for any drug delivery system, ensuring that the material does not trigger adverse immune responses, inflammation, or toxicity upon administration [29]. Nanostructured bioaerogels, derived from naturally occurring biopolymers such as chitosan, gelatin, and cellulose, offer excellent biocompatibility, making them highly suitable for biomedical applications [30]. A recent study has reported that biopolymers such as chitosan, cellulose, and starch have natural compatibility with biological systems and able to promote cell adhesion, proliferation, and minimal immune response [31]. Their ability to interact with biological tissues without causing harm is essential for maintaining therapeutic efficacy and patient safety [32]. Several studies have assessed the biocompatibility and cytotoxicity of biopolymers against different tissues and confirm their safety in medical use. Table 1 presents the illustration of studies about biopolymer-based materials in different applications.

**Figure 2:**
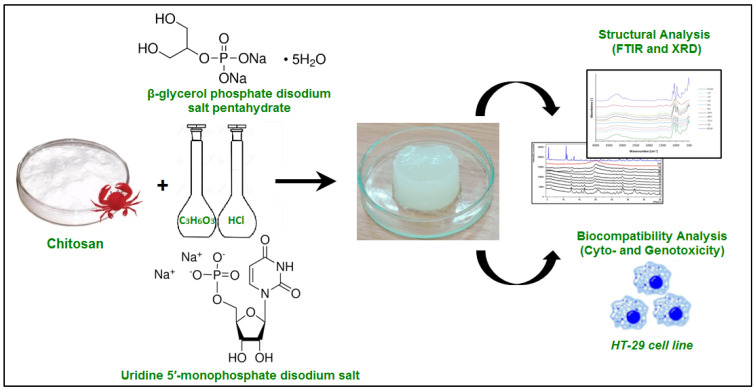
Schematic illustration of the preparation and evaluation of thermosensitive chitosan-based material subjected to structural characterization; their biocompatibility was assessed through cytotoxicity and genotoxicity tests using the HT-29 human colon adenocarcinoma cell line. Adapted with permission from [45].

In drug delivery, biocompatibility directly influences the stability and functionality of the carrier system [29]. A non-toxic, biocompatible bioaerogel ensures sustained drug release without eliciting immunogenic reactions or cytotoxic effects on healthy cells [41]. Moreover, bioaerogels can be engineered to degrade at controlled rates, aligning with the desired drug release profile [42]. This degradability minimizes the need for surgical removal of the carrier post-treatment, reducing patient discomfort and medical complications [43]. As research progresses, rigorous in vitro and in vivo studies are necessary to validate the safety and long-term compatibility of bioaerogels in various drug delivery applications. In a previous study, silica–gelatin hybrid aerogels were introduced as drug delivery devices and investigated for their biocompatibility with SCC tumour cells using a time-lapse video-microscopy technique [44]. The particles were found to be not only non-toxic but also capable of attracting migrating cells. Fluorescently labelled hybrid aerogels were synthesized by covalently binding fluorescein isothiocyanate either to the amino groups of gelatin molecules or to the amine-functionalized silica backbone prior to the sol-gel polycondensation and co-gelation steps [44]. The authors also reported that migration plots derived from time-lapse video recordings demonstrated that cells exhibited directed migration toward the aerogel particles. No signs of apoptosis or necrosis were detected at any phase of the cell cycle in the presence of the silica–gelatin aerogel particles, confirming their biocompatibility. This study demonstrated the biocompatibility of silica–gelatin hybrid aerogels, highlighting their ability to attract migrating human cells without inducing cytotoxic effects. The findings suggest their potential as safe and effective drug delivery matrices for biomedical applications. In a different study, the authors explored the modification of chitosan-based biopolymers to develop thermosensitive aerogel and hydrogel with enhanced structural and biological functionality for tissue engineering [45] (Figure 2). Using β-glycerol phosphate and uridine 5′-monophosphate as crosslinkers, they investigated structural changes through FTIR and XRD analyses, along with cytotoxicity and genotoxicity using human colon adenocarcinoma cells. The study demonstrated that the resulting materials were non-cytotoxic, non-genotoxic, and exhibited excellent biocompatibility, highlighting their promise as scaffolds for tissue regeneration.

While the initial biocompatibility and non-cytotoxicity of nanostructured bioaerogels are well documented, as we have mentioned, their long-term safety profile remains a crucial area of investigation. Abdul Khalil and co-authors mentioned that the long-term biological impact and in vivo behaviour of many constituent materials remain inadequately characterized [46]. Of particular concern is the potential for polymeric nanoparticles to induce latent cytotoxic effects, a process which may unfold over extended periods and is not detectable in short-term assays. The degradation kinetics and potential for bioaccumulation of breakdown products are key determinants of their chronic biological response [47]. Recent studies should begin to address this gap, focusing on the in vivo degradation pathways of common biopolymer-based aerogels. These investigations monitor local tissue response, systemic inflammation, and organ histology over extended periods of several weeks to months or years. Although the current body of long-term in vivo data is still expanding, the existing evidence suggests that carefully engineered bioaerogels with controlled degradation rates exhibit a favourable long-term safety profile with minimal chronic immune reaction.

## 3. Incorporation of Nanomaterials into Bioaerogels

**Figure 3:**
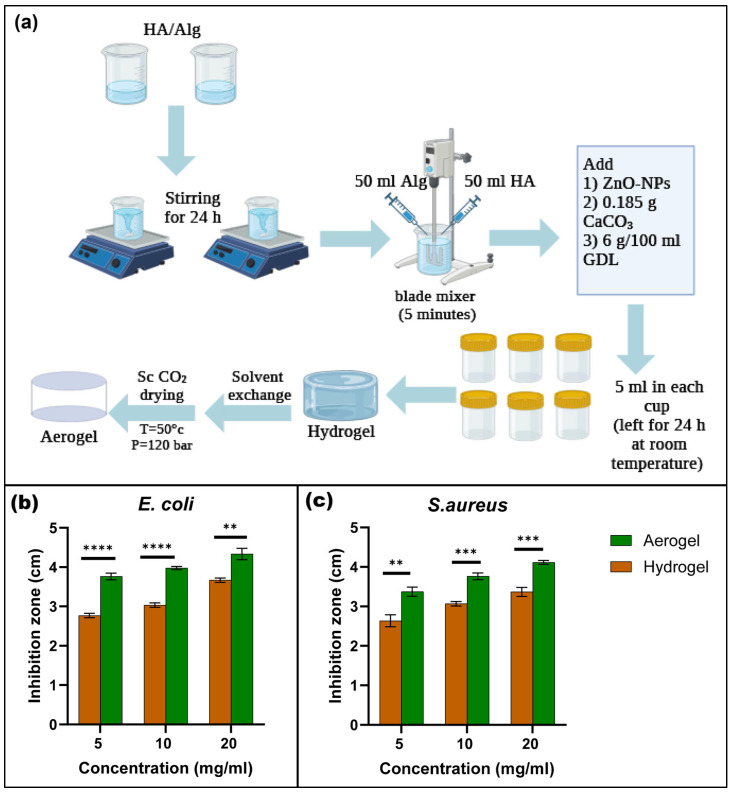
Illustration of the main steps of preparation of hyaluronic acid and alginate aerogel composites loaded with ZnO-NPs (**a**) and their antibacterial activity against *Staphylococcus aureus* (**b**) and *Escherichia coli* (**c**); Values represent mean ± standard deviation; asterisks denote statistical significance (** *p* < 0.01, *** *p* < 0.001, **** *p* < 0.0001). Adapted with permission from [59].

The integration of nanomaterials into bioaerogels has emerged as a transformative approach to enhance their structural, functional, and therapeutic properties. Nanoparticles and nanostructures provide tunable surface chemistry, high surface area, and responsive behaviors that significantly expand the application spectrum of bioaerogels in smart drug delivery systems [48,49]. Depending on the type and function of the incorporated nanomaterials, bioaerogels can be tailored to achieve controlled drug release, improved mechanical stability, enhanced bioactivity, and multifunctional responsiveness [50]. Metallic nanoparticles such as gold (Au), silver (Ag), and iron oxide (Fe_3_O_4_) are frequently embedded into biopolymer-based aerogels to confer antimicrobial, magnetic, and photothermal properties [51]. For instance, Ag nanoparticle-loaded chitosan or cellulose aerogels exhibit broad-spectrum antibacterial activity [52], while Au and Fe_3_O_4_ nanoparticles facilitate targeted or magnetically guided drug release [53,54]. Their inclusion also enhances electron conductivity, which is beneficial for biosensing and theragnostic applications. Several studies have used different metallic nanoparticles, including silver [55], gold [56], copper [57], and aluminum [58], as an antibacterial ingredient in the aerogel to enhance its properties for different biomedical applications. In a recent study, the authors loaded zinc oxide nanoparticles into hyaluronic acid and alginate aerogel to enhance its antibacterial properties for wound dressing applications [59]. The authors reported that their aerogel composite shows a promising activity against common wound pathogens, including *Staphylococcus aureus* and *Escherichia coli*, using the agar diffusion method (Figure 3). Furthermore, the results show water absorption ability of 5791 and 1585% for loaded and unloaded aerogels, respectively. The ZnO released from the aerogel exhibited a rapid release followed by a slow and sustained release.

Carbon-based nanostructures, including graphene oxide, carbon nanotubes, and carbon quantum dots, contribute to mechanical reinforcement, electrochemical responsiveness, and photothermal conversion efficiency [60]. The interaction between carbon nanostructures and the polymeric matrix generates porous, interconnected networks that enable efficient diffusion and stimuli-responsive drug release [61]. Additionally, the oxygen-containing functional groups of graphene oxide or carbon quantum dots improve biocompatibility and facilitate surface modification for ligand attachment and targeted delivery [62]. Polymeric nanocarriers, such as micelles, dendrimers, and polymeric nanoparticles, can be uniformly distributed within bioaerogel matrices to enable sustained and stimuli-responsive drug delivery [63]. Their incorporation allows the encapsulation of both hydrophilic and hydrophobic drugs, providing a dual-loading capacity that enhances therapeutic efficiency [64]. Moreover, polymeric nanocarriers improve the flexibility and degradability of the aerogel network, making them suitable for precision medicine applications such as localized cancer therapy and tissue regeneration [65]. Such nanostructured composites not only expand the functional tunability of bioaerogels but also open new pathways for personalized and on-demand therapeutic applications.

When critically compared to conventional nanocarriers such as liposomes and polymeric nanoparticles, nanostructured bioaerogels present several distinct advantages for drug delivery [66]. Their most prominent feature is an ultra-high porosity (often > 95%) and an exceptionally large specific surface area, which translates to superior drug loading capacity frequently exceeding that of solid nanoparticles by an order of magnitude [67,68]. Furthermore, their continuous three-dimensional porous network allows for more complex and tunable drug release kinetics, moving beyond the simple diffusion or erosion mechanisms of many traditional carriers [69]. Unlike liposomes, which can suffer from instability, or solid nanoparticles with limited internal volume [70], bioaerogels offer a mechanically stable, open nanostructure that can be precisely engineered for targeted delivery [71]. Finally, their composition from natural polymers (e.g., chitosan, cellulose) provides an inherent biocompatibility and potential for biodegradation that is sometimes challenging to achieve with synthetic polymer-based systems [72]. The nanostructuring of bioaerogels confers distinct advantages over their microstructured counterparts in drug delivery applications. Primarily, it drastically increases the specific surface area, leading to a substantially higher drug loading capacity [73]. Furthermore, the nanoscale pore network provides a more refined and tunable control over drug release kinetics, preventing burst release and enabling sustained delivery [74]. This nanoscale architecture also more closely mimics the natural extracellular environment, which can enhance cellular interactions, adhesion, and uptake, thereby improving therapeutic efficacy for specific tissue targets [3]. This unique combination of high payload, structural versatility, and biosafety positions nanostructured bioaerogels as a next-generation platform that complements and, in many aspects, surpasses the capabilities of existing nanocarriers.

## 4. Sensing and Controlled Release Mechanisms

**Figure 4:**
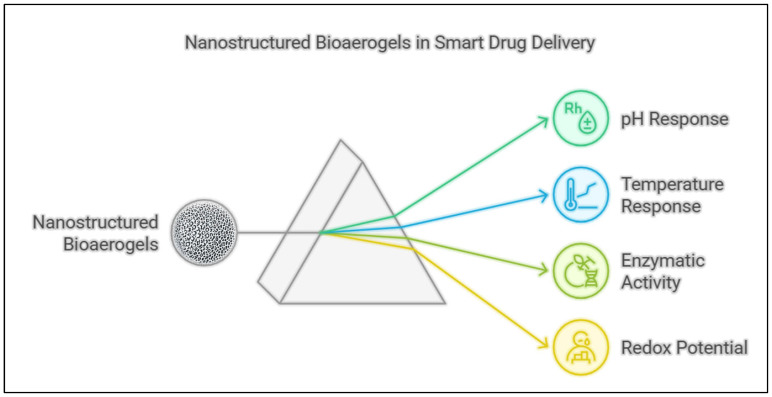
Illustration of nanostructured bioaerogel in different responsiveness for smart drug delivery.

The ability of nanostructure bioaerogels to precisely regulate drug release is a cornerstone of smart drug delivery systems, ensuring therapeutic agents are administered at the right dose, location, and time. Several researchers have explored mixing sensing and controlled release in one platform. In one study, the authors developed chitosan/reduced graphene oxide aerogel composite modified with ZrO_2_ nanoparticles as an electrochemical biosensor for luteolin detection, and they were able to successfully apply it for luteolin detection in real samples [75]. In a different study, the authors developed a biocompatible and biodegradable E-skin patch based on flexible gelatin methacryloyl aerogel for non-invasive, continuous biomarker monitoring [76]. By leveraging cryogenic treatment and controlled polymerization, the authors engineer the flexible gelatin methacryloyl aerogel structure with highly interconnected pores, endowing it with exceptional flexibility, passive cooling properties, and ultralightweight characteristics for prolonged wearability. The developed flexible gelatin methacryloyl aerogel-based E-skin was capable of simultaneously monitoring body temperature, hydration levels, and biopotentials via electrophysiological sensors while detecting glucose, lactate, and alcohol concentrations through electrochemical sensors [76]. This work introduces a novel materials strategy for next-generation E-skin platforms, offering a multifunctional, breathable, and skin-friendly solution for real-time health monitoring. Several other mechanisms have been explored by responding to specific physiological stimuli such as pH, temperature, enzymatic activity, redox potential, and others, as illustrated in Figure 4.

### 4.1. pH-Responsive Release

One of the most widely explored sensing mechanisms in bioaerogel-based drug delivery is pH-responsive release. Many pathological conditions, including cancer and infections, are associated with altered pH levels [24]. Bioaerogels functionalized with pH-sensitive polymers or linkers can selectively release drugs in acidic tumour microenvironments or inflamed tissues while remaining stable at physiological pH [24]. In one study, the authors developed a pH-responsive controlled-release bioaerogel system for antimicrobial food preservation. Cinnamaldehyde was grafted onto chitosan via the Schiff base reaction to create a pH-sensitive complex. This complex was then incorporated into bioaerogels composed of TEMPO-oxidized cellulose nanofibers and polyvinyl alcohol with varying pore structures [77]. Release experiments demonstrated that an acidic environment triggered the release of Cinnamaldehyde, with the release rate increasing from 10.3% to 68.4% as pH rose. In a different study, the authors developed a pH-responsive composite bioaerogel for controlled drug release by combining collagen and TEMPO-oxidized cellulose nanofibers [78]. They utilized the self-assembly behaviour of COL molecules to form a physically cross-linked supramolecular double network with cCNFs, enhancing the bioaerogel’s structural integrity. To further improve stability and slow-release capabilities, cyclodextrins were incorporated into the composite. The authors used 5-fluorouracil as a model drug; they demonstrated that the bioaerogel exhibited pH-sensitive drug release, showing superior slow-release performance [78]. Similarly, in a different study, the authors developed pH-responsive collagen composite aerogels by modifying cellulose nanofibrils through TEMPO oxidation and amination with diethylenetriamine [79]. These functionalized nanocellulose were then combined with collagen using self-assembly pretreatment and directional freeze-drying, resulting in layered-oriented double network structures. 5-fluorouracil (5-FU) was also used as a model drug to analyse the pH-triggered drug release mechanism. The findings showed that the bioaerogels had excellent pH-sensitive drug release behaviour [79]. The incorporation of functionalized biopolymers, such as modified cellulose nanofibrils and collagen, enhances the structural stability, drug retention, and responsiveness of these materials. Such advancements are particularly valuable in biomedicine and tissue engineering, where precise and sustained drug release is essential for improving therapeutic outcomes and minimizing side effects.

**Figure 5:**
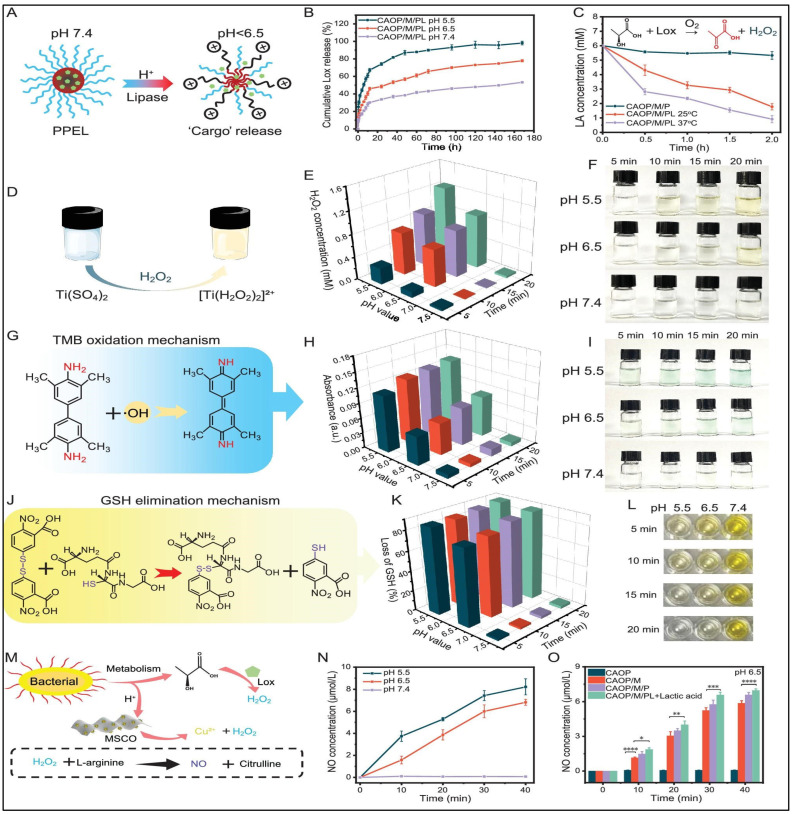
Schematic illustration of functional characterization of a smart hydrogel system. (**A**) pH/lipase-triggered drug release mechanism and (**B**) cumulative release profile. (**C**) Lactic acid scavenging. (**D**–**I**) Mechanisms and validation of self-supplying H_2_O_2_ and generating •OH. (**J**–**L**) Glutathione (GSH) depletion. (**M**–**O**) Bacteria-responsive nitric oxide (NO) cascade release. Data are mean ± SD; * *p* < 0.05, ** *p* < 0.01, *** *p* < 0.001, **** *p* < 0.0001. Adapted with permission from [82].

One of the most beneficial applications of pH responsiveness is oral administration of insulin, which has been extensively explored through various innovative delivery strategies. Researchers have developed pH-responsive methacrylic acid-based aerogels incorporating microcrystalline cellulose for oral insulin delivery [80]. Using free radical polymerization, they synthesized aerogels that exhibited significant pH-sensitive swelling, protecting insulin in acidic environments while enabling controlled release in the intestines. While in vitro studies demonstrated lower insulin release at pH 1.2 and enhanced release at pH 6.8. Further in vivo experiments in diabetic Wistar rats showed a sustained hypoglycaemic effect for 24 h, confirming their potential for prolonged insulin delivery [80]. In another study, Alibolandi and co-workers developed a dextran-based polymersome for insulin encapsulation, assessing its performance through both in vitro and in vivo studies [81]. The pH-responsive polymersome was synthesized via a direct hydration method, incorporating a copolymer blend with an aqueous insulin solution at ambient temperature. Insulin release was evaluated under different pH conditions (1.2 and 7.4) and in diabetic rat models. The system demonstrated an encapsulation efficiency exceeding 90%, with minimal insulin leakage in the acidic gastric environment and significant release in simulated intestinal conditions, ensuring optimal permeability. In vivo findings revealed a pronounced hypoglycaemic effect in diabetic rats, underscoring the potential of such biopolymer-based carriers for effective oral insulin administration. In a different study, the authors prepared a cascading self-activating antibacterial aerogel capable of responding to multiple bacterial infection cues within the wound microenvironment, thereby exhibiting on-demand antibacterial activity [82]. The system exploited lactic acid, a metabolic byproduct responsible for local pH reduction, as a trigger for lactate oxidase (Lox) activation to generate pyruvate and H_2_O_2,_ key intermediates for reactive oxygen species (ROS) formation (Figure 5). To achieve controlled enzyme delivery, the researchers encapsulated Lox-loaded PPEL micelles within a hydrogel matrix that remained stable under physiological conditions but disintegrated in response to acidic pH or lipase activity. This design enabled pH- and enzyme-induced release of Lox, with release efficiencies increasing from 53.2% at pH 7.4 to 98.1% at pH 5.5, confirming the aerogel’s strong responsiveness to infection-related acidity. Moreover, Lox released from the aerogel effectively decomposed lactic acid from 6 mM to 0.92 mM at 37 °C, validating the system’s capacity for self-triggered, bacteria-mediated activation and metabolic regulation.

### 4.2. Temperature-Responsive Release

**Figure 6:**
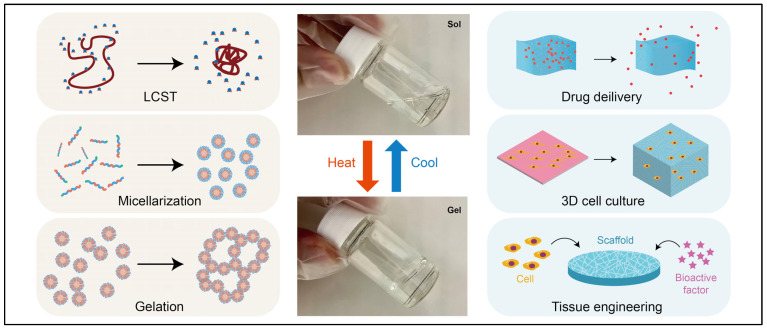
Schematic illustration of temperature-responsive bioaerogels showing different mechanisms and applications of temperature-responsiveness. Adapted with permission from [86].

Temperature-responsive bioaerogels have also been developed to exploit the slight temperature variations in diseased tissues. These systems undergo conformational changes or phase transitions at predetermined temperatures, enabling controlled drug release [83]. In one study, the authors developed an intelligent temperature-responsive bioaerogel reactor by combining thermosensitive molecularly imprinted polymers with composite bioaerogels for the efficient enrichment of ursolic acid. The bioaerogel exhibited an exceptional ability to spontaneously adsorb and desorb ursolic acid by adjusting the reaction temperature [84]. After optimizing the synthesis conditions, the authors reported that the aerogel was successfully applied to lingonberry (*Vaccinium vitis-idaea* L.) extracts, demonstrating its potential as an intelligent, temperature-controlled adsorption material for solid-phase extraction of triterpenoid acids in natural products. Thermoresponsive aerogels or thermogels exhibit a sol-gel transition upon temperature elevation, making them valuable biomaterials for various biomedical applications [85]. This phase transition arises from multiscale thermoresponsive mechanisms, including lower critical solution temperature (LCST) behaviour, micellization, and micelle aggregation (Figure 5). Owing to their tunable phase conversion, thermogels remain injectable in solution form while undergoing in situ gelation within the body, facilitating localized and sustained therapeutic delivery [86]. Their ability to form hydrogels and aerogels at physiological temperatures makes them particularly attractive for biomedical applications. Figure 6 illustrates the phase transition mechanisms of temperature-responsive gels, including lower critical solution temperature transition, micellization, and gelation.

### 4.3. Enzyme-Responsive Release

**Figure 7:**
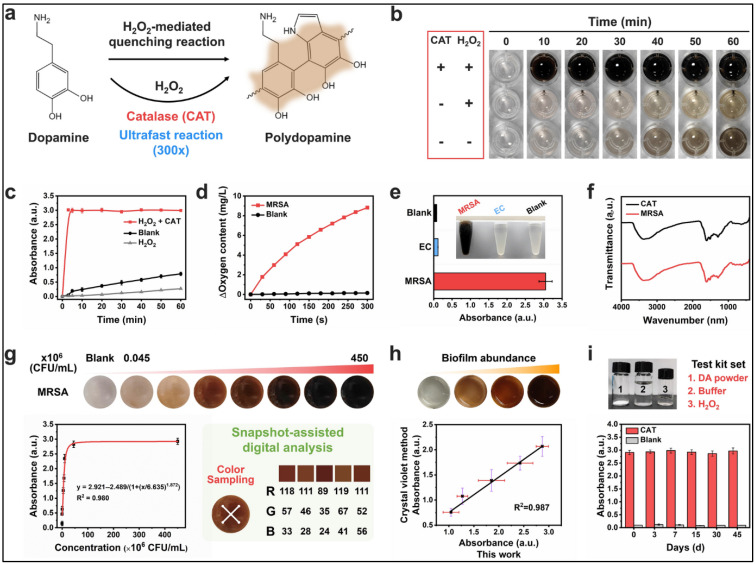
Ultrafast polymerization reaction-based detection of SA. (**a**) Schematic representation of the dopamine polymerization reaction catalysed by H_2_O_2_ and catalase, forming polydopamine. (**b**) Time-lapse images of dopamine polymerization under different conditions. (**c**) Absorbance measurements at OD652 showing reaction kinetics with various treatments. (**d**) Changes in oxygen content of H_2_O_2_ solution upon MRSA addition, recorded using a dissolved oxygen analyser. (**e**) Visual and spectroscopic detection of MRSA, with *E. coli* as a control. (**f**) FTIR spectra of polymerization products catalysed by MRSA and CAT. (**g**) Detection range of the MRSA-responsive polymerization reaction, validated through snapshot-assisted digital analysis. (**h**) Comparison of the proposed method with the conventional crystal violet staining for MRSA biofilm quantification. (**i**) Independent test kit for MRSA detection, demonstrating repeatability over multiple days. Adapted with permission from [94].

Enzyme-responsive nanostructured bioaerogels have emerged as a promising class of smart drug delivery carriers due to their ability to provide targeted and controlled drug release in response to specific enzymatic activity [87]. Enzymes play a crucial role in various biological processes and are often overexpressed in pathological conditions such as cancer, inflammation, and bacterial infections [88]. By leveraging enzyme-sensitive linkages within bioaerogels, therapeutic agents can be selectively released at the site of disease, minimizing systemic toxicity and enhancing therapeutic efficacy [89]. One study developed a dual-responsive active packaging system for controlled antimicrobial release in food preservation [90]. Zeolitic imidazolate framework-8 nanoparticles were grown in situ on TEMPO-oxidized cellulose nanofiber films and loaded with carvacrol, followed by the electrostatic adsorption of pectin as a “gatekeeper.” The resulting system exhibited both pH- and enzyme-responsive release mechanisms, reacting to acidic conditions (pH 5.0) and pectinase produced by microorganisms [90]. The design of enzyme-responsive bioaerogels typically involves the incorporation of biodegradable polymeric networks or functional moieties that undergo cleavage in the presence of disease-associated enzymes [91]. Matrix metalloproteinase-sensitive bioaerogels have been extensively studied for cancer therapy, as these enzymes are upregulated in the tumour microenvironment, facilitating localized drug release [92,93]. A recent study presents an innovative and multifunctional bioaerogel system designed for the rapid detection and treatment of *Staphylococcus aureus* (SA) infections, addressing the critical challenge of timely wound monitoring and intervention [94]. The authors integrate the bioaerogel with hydrogen peroxide (H_2_O_2_), dopamine, and GelMA polymer, leveraging an enzyme-responsive detection mechanism. The system detection strategy capitalizes on SA-secreted catalase, which decomposes H_2_O_2_ into oxygen (Figure 7). This oxygen generation accelerates the polymerization of dopamine into polydopamine (PDA), leading to a rapid colorimetric change from colourless to deep brown. The bacterial detection process is remarkably fast, completing within 10 min, and offers high sensitivity. Additionally, the results are visually distinguishable by the naked eye and can be further quantified using a smartphone-based digital analysis system, making it a practical and accessible tool for infection monitoring. Beyond detection, the system incorporates a dual antibacterial mechanism. The in situ-generated PDA enables both chemical antibacterial action and photothermal therapy, significantly enhancing bacterial clearance [94]. The same authors reported that in vivo animal studies further validate the system’s therapeutic efficacy, demonstrating its ability to monitor and eradicate bacteria, reduce inflammation, promote collagen deposition, and accelerate wound healing. This study presents a promising enzyme-responsive aerogel system for the rapid detection and treatment of SA infections. Its ability to visually detect bacteria within 10 min, coupled with dual antibacterial mechanisms, enhances both diagnostic efficiency and therapeutic effectiveness. The multifunctional design holds great potential for advanced wound care applications.

### 4.4. Other Responsive Release

**Figure 8:**
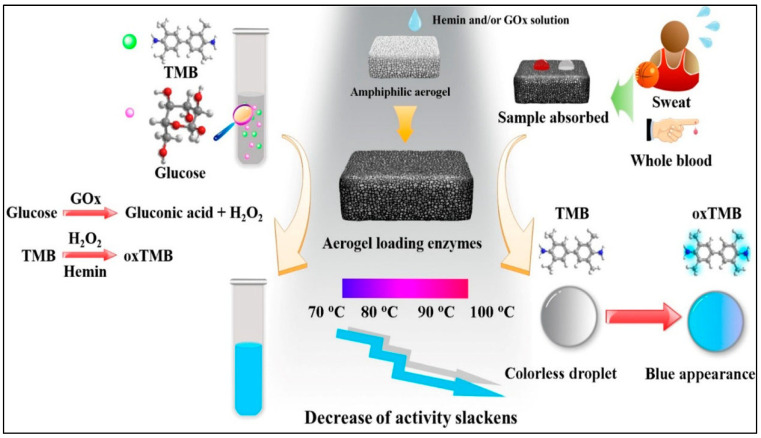
Schematic illustration of the enzyme-immobilized aerogel for glucose sensing. The aerogel matrix facilitates the co-immobilization of glucose oxidase (GOx) and hemin. Adapted with permission from [100].

Beyond passive stimulus-responsive systems, externally triggered release mechanisms, such as magnetic, ultrasound, and light-responsive bioaerogels, offer another level of precision in drug administration [95,96]. These approaches allow for real-time control over drug release, making them particularly valuable for personalized medicine and on-demand therapeutic interventions [97]. Enzyme immobilization has gained significant attention in biocatalysis, biosensors, and industrial applications due to its ability to enhance enzyme stability and reusability [98]. However, free-floating enzymes often suffer from deactivation under harsh conditions, such as high temperatures, limiting their practical applications [99]. To address this challenge, researchers have explored various immobilization strategies, including the use of nanostructured materials and functionalized aerogels, to provide a stable microenvironment for enzyme retention and activity [100]. The authors investigated an amphiphilic poly(vinyl alcohol)-based aerogel as a substrate for multienzyme immobilization, aiming to enhance enzyme stability under extreme conditions. The aerogel, modified with maleic acid, improved surface wettability, facilitating efficient enzyme loading and stabilization (Figure 8). By co-immobilizing glucose oxidase and hemin within the aerogel matrix, the system successfully catalysed glucose oxidation and 3,3,5,5-tetramethylbenzidine oxidation while maintaining enzymatic activity at elevated temperatures (70–100 °C). The aerogel–enzyme composite demonstrated remarkable thermal stability, as evidenced by the reduced rate of enzymatic degradation under prolonged exposure to high temperatures. Furthermore, the modified aerogel was utilized as a glucose sensor, enabling accurate glucose detection in whole blood and sweat [100]. The composite material exhibited excellent adsorption capacity, catalytic efficiency, and mechanical durability, making it a promising candidate for biosensing applications. The study highlighted the potential of enzyme-loaded aerogels as robust platforms for biocatalytic processes and biosensors, offering enhanced enzymatic stability and functionality under extreme environmental conditions.

## 5. Targeting Strategies: Active vs. Passive Targeting

**Figure 9:**
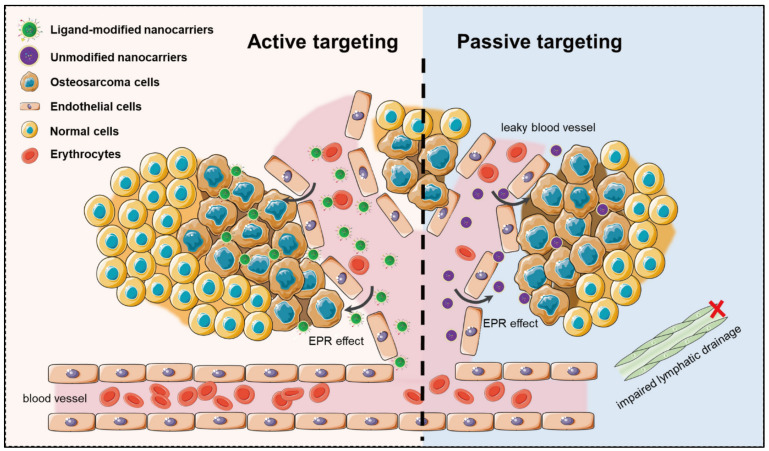
Schematic drawing of nano-delivery in anti-tumour therapy: passive targeting relies on the enhanced permeability and retention effect for tumour accumulation, while active targeting uses ligand-modified nanocarriers for receptor-mediated drug delivery. Adapted with permission from [101].

Nanostructured bioaerogels offer a versatile platform for controlled drug delivery, and their targeting capabilities can be categorized into passive and active targeting mechanisms [101]. These approaches enable the precise delivery of therapeutic agents to specific tissues or diseased sites, improving drug bioavailability and reducing systemic toxicity. Passive targeting of bioaerogels exploits the inherent physiological characteristics of diseased tissues, particularly the enhanced permeability and retention effect observed in tumours and inflamed tissues [102]. Due to their highly porous architecture and nanoscale dimensions, bioaerogels can accumulate preferentially at pathological sites where vascular permeability is increased [103]. This phenomenon enables the gradual accumulation of drug-loaded bioaerogels in tumour tissues, leading to prolonged therapeutic effects [12,104]. Additionally, the biodegradability and hydrophilic nature of bioaerogels contribute to their extended circulation time, further enhancing their passive targeting efficiency [50]. However, the success of passive targeting is often limited by heterogeneity in tumour vasculature and poor penetration in dense tissues, necessitating more precise delivery mechanisms [105]. Active Targeting of Bioaerogels enhances the specificity of drug delivery by functionalizing bioaerogels with targeting ligands such as antibodies, peptides, aptamers, or small molecules that bind selectively to receptors overexpressed on diseased cells [24]. Folate-conjugated bioaerogels can target cancer cells that exhibit high folate receptor expression, while transferrin-modified aerogels enhance drug delivery to iron-regulated tumour cells [106]. This ligand–receptor interaction facilitates cell-specific internalization of bioaerogels, ensuring more effective drug accumulation within targeted cells compared to passive mechanisms. Figure 9 illustrates the difference between active and passive targeting of bioaerogels in drug delivery.

By combining targeting ligands with stimuli-responsive elements of bioaerogels, these systems achieve a dual-targeting effect, improving therapeutic outcomes and reducing side effects [24]. Despite the advantages of active and passive targeting strategies, several challenges remain in their clinical translation. Passive targeting is often affected by variations in vascularization, while active targeting depends on ligand stability, receptor expression heterogeneity, and immune system interactions. Future advancements in bioconjugation techniques, hybrid bioaerogel formulations, and personalized medicine approaches will be crucial in optimizing targeting efficiency and broadening the applicability of nanostructured bioaerogels in drug delivery. The therapeutic component of bioaerogels comprises the actual drug or therapeutic agents intended for the treatment or management of a specific disease [107]. The design of these systems focuses on maximizing the therapeutic index while ensuring stability and compatibility with the other system components.

## 6. Challenges and Limitations of Nanostructured Bioaerogels in Smart Drug Delivery

Nanostructured bioaerogels in smart drug delivery have emerged as a revolutionary approach in the realm of targeted therapies, offering unprecedented precision in drug delivery and controlled release mechanisms [24,108]. Despite their potential to transform medical treatment, several challenges and limitations still hinder their widespread adoption and effectiveness. These challenges span across technical, biological, regulatory, and commercial aspects, each of which requires comprehensive consideration to realize the full potential of bioaerogels. One of the primary technical challenges facing the development of bioaerogels is the complexity of system design. Designing a drug delivery system that can accurately detect environmental stimuli and respond appropriately involves intricate engineering [49]. The integration of sensors, actuators, and drug reservoirs into a single, often microscopic, platform necessitates advanced materials science, microfabrication, and nanotechnology expertise [109]. Additionally, these components must be perfectly synchronized to function in unison, which adds another layer of complexity to the design and manufacturing process.

Material selection poses another significant hurdle. The materials used must not only be biocompatible and biodegradable but also possess the appropriate mechanical strength, flexibility, and degradation rates suitable for their intended application [110]. Furthermore, these materials must be stable under physiological conditions and during storage, and they must not degrade into toxic byproducts. Developing materials that meet all these criteria remains a daunting task for researchers and manufacturers alike. Moreover, the long-term effects of these materials and their degradation products on the body and the environment are not always fully understood, which complicates safety assessments. The complexity of the biological environment also presents significant challenges. The human body is a highly dynamic system with complex biochemical pathways that can interfere with the functioning of drug delivery systems.

The high cost of research, development, and regulatory compliance poses substantial commercial challenges for the widespread adoption of bioaerogels in smart drug delivery. The materials, technologies, and expertise required for the development of such systems are expensive, and the return on investment can be uncertain, especially if the final product does not reach the market or achieve widespread adoption. Market acceptance and adoption also pose significant hurdles. Healthcare providers and patients may be hesitant to adopt new technologies, particularly those that significantly deviate from traditional treatment modalities. Overcoming this scepticism requires not only demonstrating the clear benefits of bioaerogels in drug delivery over existing therapies but also ensuring the systems are easy to use and integrate into current medical practices.

## 7. Conclusions and Future Perspectives

Multimodal nanostructured bioaerogels represent a paradigm shift in the landscape of smart drug delivery systems, moving beyond conventional carriers by integrating therapeutic, targeting, and diagnostic functions into a single, sophisticated platform. Their exceptional properties, primarily stemming from their natural polymer base, establish them as an ideal matrix for advanced biomedical applications. The ability to further functionalize bioaerogels with nanomaterials such as metallic nanoparticles or carbon-based structures enhances their functionality, enabling externally triggered release. Looking forward, the clinical translation of this promising technology hinges on addressing several interconnected challenges and strategically embracing emerging opportunities. A primary hurdle remains the scalability and economic viability of manufacturing processes. While laboratory-scale synthesis, particularly supercritical drying, is effective for producing high-quality aerogels, adapting these methods for cost-effective, high-throughput industrial production is a critical next step. Innovations in ambient pressure drying or the development of continuous-flow processes could be pivotal in overcoming this bottleneck, making these advanced materials accessible for widespread pharmaceutical use. Furthermore, while the short-term biocompatibility and non-cytotoxicity of biopolymer-based aerogels are well-supported by existing literature, a more rigorous investigation into their long-term in vivo safety profile is imperative. Future research must prioritize longitudinal studies that meticulously track the degradation kinetics of these nanostructured materials, the biological fate of their breakdown products, and any potential for chronic inflammatory responses or off-target accumulation. Establishing a comprehensive safety database will be non-negotiable for regulatory approval and building clinical confidence.

The complexity of optimizing bioaerogel synthesis and performance presents a perfect opportunity for the integration of data-driven approaches like machine learning and artificial intelligence. Machine learning algorithms can analyse vast, multi-factorial datasets to predict relationships between synthesis parameters (e.g., precursor concentration, cross-linking density) and the resulting structural and functional properties of the aerogel. This can dramatically accelerate the rational design of bespoke bioaerogels, optimizing them for specific drug encapsulation efficiencies, tailored release profiles, and targeted interactions with particular cell types, thereby reducing the traditional trial-and-error timeline in material development. Finally, the convergence of bioaerogels with 3D bioprinting technologies opens a new frontier in personalized medicine. The combination of the bioaerogels’ biomimetic porosity and drug reservoir capabilities with the spatial precision of 3D printing allows for the fabrication of complex, patient-specific tissue engineering scaffolds and implants. These constructs could provide not only structural support for tissue regeneration but also sustained, localized delivery of multiple growth factors or therapeutic agents, creating dynamic environments that actively guide the healing process. By systematically addressing the challenges of scalable manufacturing and long-term biosafety, while simultaneously harnessing the power of computational design and advanced fabrication, nanostructured bioaerogels are poised to transition from a revolutionary research concept to a cornerstone of next-generation, personalized precision medicine, ultimately offering more effective, safe, and adaptable therapeutic solutions.

## Figures and Tables

**Table 1:** Illustrations of studies on biocompatibility and cytotoxicity of bioaerogels prepared from different precursor materials.

Precursor Material/s	Form of Material	Preparation	Type of Cells	Remark	References
Silk fibroin and chitosan	Aerogel scaffolds	Lyophilization	MC3T3-E1 cells	Promotes osteogenic differentiation in the cells	[33]
Methylcellulose and bacterial nanocellulose	Aerogel	3D printing	NIH/3T3 fibroblast cells	Biocompatibility and no sign of toxicity	[34]
Gelatin and silica	Hybrid aerogels	Supercritical drying	Osteoblasts cells	Positive effect on cell growth	[35]
Starch and chitosan	Composite aerogels	Solvent exchange	Intestinal Caco-2	No sign of toxicity	[36]
Collagen	Aerogels	Lyophilization	Mouse fibroblast	Consistent pattern of elongation and proliferation	[37]
Nanocellulose and chitosan	Bioaerogel	Freeze drying	L929 fibroblast cell	Enhanced the proliferation of the cells	[38]
Silica, silk, and chitosan	Hybrid aerogels	3D printing	L929 fibroblast cell	Confirmed the aerogel’s full biocompatibility with the cells	[39]
Nanofibrillated cellulose/glucosamine	Aerogel implant	3D printing	MG-63 cells	Significant increase in MG-63 proliferation	[40]

## Data Availability

Not applicable.
